# Validity and reliability of capillary vs. Venous blood for the assessment of haemoglobin mass and intravascular volumes

**DOI:** 10.3389/fphys.2022.1021588

**Published:** 2022-11-24

**Authors:** Joshua T. Royal, Jason T. Fisher, Tinkara Mlinar, Igor B. Mekjavic, Adam C. McDonnell

**Affiliations:** ^1^ Department of Automation, Biocybernetics, and Robotics, Jozef Stefan Institute, Ljubljana, Slovenia; ^2^ Jozef Stefan International Postgraduate School, Ljubljana, Slovenia; ^3^ Department of Biomedical Physiology and Kinesiology, Simon Fraser University Burnaby, Burnaby, BC, Canada

**Keywords:** haemoglobin mass, CO rebreathing, validity, venous blood, capillary blood

## Abstract

**Objectives:** Haemoglobin mass (Hbmass) assessment with the carbon monoxide rebreathing method is a more accurate estimate than other measures of oxygen-carrying capacity. Blood may be collected by several means and differences in the measured variables may exist as a result. The present study assessed the validity and reliability of calculated Hbmass and intravascular volumes obtained from capillary blood (CAP) when compared to venous blood (VEN) draws.

**Methods:** Twenty-two adults performed a carbon monoxide rebreathing procedure with paired VEN and CAP draws at baseline, pre-rebreathing and post-rebreathing (POST). Thirteen of these participants performed this protocol on two occasions to assess the data reliability from both blood sampling sites. In a second experiment, 14 adults performed a 20-min seated and a 20-min supine rest to assess for the effect of posture on haematological parameters.

**Results:** Haemoglobin mass (CAP = 948.8 ± 156.8 g; VEN = 943.4 ± 157.3 g, *p* = 0.108) and intravascular volume (CAP = 6.5 ± 1 L; VEN = 6.5 ± 0.9 L, *p* = 0.752) were statistically indifferent, had low bias (Hbmass bias = 14.45 ± 40.42 g, LoA -64.78 g—93.67 g) and were highly correlated between sampling techniques. Reliability analysis demonstrated no difference in the mean change in variables calculated from both sampling sites and good to excellent intraclass correlation coefficients (>0.700), however, typical measurement error was larger in variables measured using CAP (VEN Hbmass TE% = 2.1%, CAP Hbmass TE% = 5.5%). The results indicate that a supine rest prior to the rebreathing protocol would have a significant effect on haemoglobin concentration and haematocrit values compared to a seated rest, with no effect on carboxyhaemoglobin %.

**Conclusion:** The present study demonstrates that CAP and VEN were comparable for the calculation of Hbmass and intravascular volumes in terms of accuracy. However, reduced reliability and increased error in the CAP variables indicates that there are methodological considerations to address when deciding which blood drawing technique to utilise. To reduce this CAP error, increased replicate analyses are required.

## 1 Introduction

Measurements of total haemoglobin mass (Hbmass; g or g·kg^−1^), red blood cell volume (RBCV), total blood volume (BV) and plasma volume (PV) may be used to monitor an individual’s health status (Otto et al., 2017). Hbmass calculation offers an insight into absolute blood oxygen-carrying capacity and is often used to examine the effects of exercise training interventions (Heinicke et al., 2001; Rønnestad et al., 2021), hypoxic exposures (Pugh, 1964; Hahn et al., 2001) and altitude training (Gore et al., 1998; Schmidt and Prommer, 2008; Gough et al., 2012). Hbmass is more accurate and thus more informative than other measures like haematocrit (Hct) or haemoglobin concentration ([Hb]; g·mL^−1^), which may potentially misrepresent actual content as a consequence of posture and hydration status changes (Dill and Costill, 1974; Durussel et al., 2013).

Venepunctures require a phlebotomist in order to obtain a successful blood draw. While all blood drawing techniques carry an inherent risk, the likelihood of a vasovagal reaction is approximately 1% and lower with capillary draws (WHO, 2010). This risk is further reduced with repeat visits. Capillary blood draws are often regarded as a cost-effective, quick and simple blood draw method and often less painful than a venepuncture. While easier in theory for the layperson to conduct, the fingerstick lancing technique draws blood from the capillaries, venules and arterioles, as well as interstitial fluid and may lead to a haemolysed sample. However, such contamination of the blood sampled may be reduced with good blood flow and by eliminating the first blood drop of the draw (Krleza et al., 2015). Comparisons between venous and capillary blood for a multitude of variables have been made over the last 100 years (Duke and Stofer, 1922; Andresen and Mugrage, 1938; Mohammed et al., 2010; Fliervoet et al., 2022; Kellenberger et al., 2022). Haemoglobin concentration and Hct values in adults and children are typically higher when the sample is drawn from the capillary rather than the vein (Moe, 1970; Hütler et al., 2000; Neufeld et al., 2002), which highlights a potential risk of using capillary and venous blood interchangeably. Various iterations of the carbon monoxide (CO) rebreathing method are frequently implemented (Table 1). Each iteration of the protocol has details which differ between research groups. Commonly, these are the rest period prior to rebreathing, the rebreathing time itself, dosage of CO, the timing of blood sampling, the sampling technique (capillary or venous), the number of replicate analyses and posture.

**TABLE 1:** Previous iterations of the CO rebreathing protocol utilised for the assessment of Hbmass and intravascular volumes.

#	Citation	Rebreathing protocol					Blood sampling				
		Rest period (min)	Position	Pre-breathing 100% O_2_ (min)	CO dose (ml)	Rebreathing time (min)	Sampling location	Timing (min)	Replicate analysis	Blood analyser	Coefficient of variation (%)
**1**	Thomsen et al. (1991)	15	supine	2	50	10	Antecubital vein from each side of the body (2 × 2 ml)	Pre, 1, 2, 3, 4, 5, 6, 7, 8, 10, 12, 15	2 x sample	Radiometer OSM3	2.3%–2.6% (Hb)
**2**	Burge and Skinner, (1995)	20	seated	4	50–90	10	Antecubital vein (2 ml)	Pre (2 samples), 10 min (single post sample)	4–6 x per sample	Radiometer OSM3	CoV: 0.8%
**3**	Hütler et al. (2000)	15	seated	none	1 ml kg^−1^ BW + 10 ml (100 ml max)	Continued until plateau or decrease in COHb% observed in CAP and VEN	VEN: Antecubital vein (1 ml) CAP: earlobe (volume not indicated)	VEN: Pre, 2, 4, 6, 8, 10, 12 min. CAP: Pre, 1, 3, 5, 7, 9, 11 min	3 x per sample	Radiometer OSM3	VEN: 3.0 ± 1.3% (Hbmass); 3.6 ± 1.5% (RBCV); PV: 5.2 ± 2.6% (PV) CAP: 3.3 ± 1.6% (Hbmass); 3.6 ± 1.8% (RBCV); PV: 5.1 ± 9.8% (PV)
**4**	(Schmidt and Prommer, 2005; Prommer and Schmidt, 2007)	15	seated	none	1 ml kg^−1^ BW (99.5% CO)	CO bolus followed by rebreathing of 3.5 L O_2_ for 2 min	VEN: Antecubital vein (∼2 ml) CAP: earlobe (85 µl)	Pre (-1 min), 1, 2, 4, 6, 8, 10, 12.5, 15	No replicate info, 1 x per sample Study II, duplicate [Hb] only	Radiometer ABL 520	1.7%–2% TE%
**5**	Siebenmann et al. (2017)	20 (500 ml water)	supine with legs elevated	4	1.0 ml kg^−1^ BW (f) 1.5 ml kg^−1^ BW (m) (99.997% CO)	10	Antecubital vein (2 ml heparinised syringe)	Pre rebreathing, 10 min	4 x per sample	Radiometer ABL 800 Hct micromethod	1.49% TE%
**6**	Oberholzer et al. (2020)	20 (500 ml water)	supine with legs elevated	4	0.5–1.5 ml kg^−1^ BW 99.997% CO	10	2 ml	Pre (0 min), 6, 8, 10 min	4 x per sample	Radiometer ABL 800 Hct micromethod	1.5%–6.5% TE%
**7**	Kellenberger et al. (2022)	15	seated	none	1.0 ml kg^−1^ BW (f) 1.2 ml kg^−1^ BW (m) (100 ml max)	2	CAP: earlobe 3 at baseline, Single sample after that (*cf.* Figure 1.)	Pre (0min), 2, 4, 6, 8, 10, 12 min	1 x each of three capillary samples at baseline 1 x per other samples	Radiometer ABL 800	N/A
		20	supine	1	1.0 ml kg^−1^ BW (f) 1.2 ml kg^−1^ BW (m)	10	VEN: Antecubital vein (2 ml heparinised syringe) CAP: earlobe 3 at baseline, Single sample after that (*cf.* Figure 1.)	Pre (0min), 6, 8, 10 min	2 x per venous sample 1 x each of 3 capillary samples at baseline 1 x other samples	Radiometer ABL 800	N/A

Note: VEN, venous blood; CAP, capillary blood; BW, body weight; CO, carbon monoxide; COHb%, Carboxyhaemoglobin; O_2_.oxygen.

The influence of posture upon blood values is widely reported (Maughan et al., 2001; Lippi et al., 2015; Astolfi et al., 2020). Despite this, one major discrepancy between protocols is the posture assumed prior to and during CO rebreathing, for example, a seated position is utilised in the Schmidt and Prommer (2005) and Hütler et al. (2000) methods, whereas in the Siebenmann et al. (2017) and Burge and Skinner (1995) methods, a supine position, with or without legs raised was assumed, respectively. To investigate this, Durussel et al. (2013) conducted a postural analysis prior to performing the “Schmidt and Prommer” CO rebreathing method. Participants assumed a supine position for 10 min before moving to a seated position for a further 10 min, with venous blood draws after each stage. No significance was found in [Hb], Hct or resultant intravascular volume values as a result of the participant’s posture. However, critically, there are other works (Hagan et al., 1978; Diaz et al., 1979) that indicate plasma volume expansion occurs in the supine position as a result of a cephalad fluid shift when moving from upright to supine, which should be considered. In order to further clarify this matter, the present study aims to replicate the idea behind the work of Durussel et al. (2013) whilst altering the protocol. Firstly, by measuring a baseline value immediately prior to the first posture and secondly, by increasing the participants’ time spent in each posture. Namely, 20 min compared to 10 min was chosen as this is the length of time recommended to maintain the supine position in the protocol chosen for the present manuscript (Siebenmann et al., 2017) and critically, it is longer than the recommended duration (>15 min) to allow vascular volumes to stabilise (Tan et al., 1973; Smith et al., 1994).

Finally, Durussel et al. (2013) performed the CO rebreathing protocol described by (Prommer and Schmidt, 2007) (Table 1) and found that the carboxyhaemoglobin saturation levels (COHb%) at baseline (BL) and at 8 min after the onset of the 2-min CO rebreathing (6 min post-CO rebreathing), from capillary blood to be consistently higher than those estimated from venous blood. However, importantly, no significant difference in the ΔCOHb% (Post COHb%—Pre COHb%) obtained by either method was noted. Good repeatability was found in Hbmass determination from both capillary and venous blood samples, which supports other research groups’ findings using this protocol (Gore et al., 2006; Prommer and Schmidt, 2007). Hütler et al. (2000) reported that despite finding significantly higher [Hb] and Hct values in capillary blood samples, these variables were highly correlated across sampling techniques ([Hb]: *r* = 0.90, Hct: *r* = 0.93). Additionally, they noted that ΔCOHb% (10th min—0 min; CAP = 6.24 ± 0.59%, VEN = 6.26 ± 0.60%) and Hbmass values (959 ± 106 g vs. 962 ± 110 g; *r* = 0.987) were statistically indifferent across sampling techniques.

Finally, Siebenmann et al. (2017) utilised the micromethod to assess Hct prior to rebreathing and obtain the other values pertinent to calculating Hbmass from venous blood. Therefore, research comparing Hbmass and intravascular volume calculated from only capillary or venous blood samples, while conducting the supine CO rebreathing method described by Siebenmann et al. (2017) is scarce. Kellenberger et al. (2022) performed a comparison between capillary and venous blood, however, a test-retest reliability analysis, which would reflect the typical measurement error, is crucially missing from their analysis.

Therefore, the aims of the present study were as follows:

• Compare the validity and reliability of values obtained from capillary blood draws compared to venous blood draws.
• Assess the effect of posture (seated vs. supine) upon haematological values relevant to Hbmass assessment prior to the start of a CO rebreathing protocol.
• Provide practical implications of the present study for clarity for researchers considering the assessment of Hbmass and intravascular volumes.

## 2 Methods

The participants’ written informed consent was obtained prior to the study, at which point they were advised that they were free to withdraw from the study and/or terminate a trial at any time. The procedures were approved by the National Committee for Medical Ethics at the Ministry of Health (Republic of Slovenia; approval number: 0120-401/2020/8) and conformed to the standards set by the Declaration of Helsinki, except for registration in a database.

### 2.1 Experiment 1: Validity and reliability

Paired venous and capillary blood draws were analysed from 22 adults (18 males, 4 females) aged 29.1 ± 6.4 years. Of the 22 participants, 13 performed the experiment twice to assess the reliability of both blood drawing techniques. These repeat measurements were conducted at the same time of the day, separated by a minimum of 48 h and a maximum of 1 week. Participant inclusion criteria were: 18 years of age or older, non-smoker and without any diagnosis of a medical condition. Twenty-six participants were initially included, however, four were excluded due to the loss of blood samples as a result of clotting, reduced or no blood flow and as such could not be included in the analysis.

#### 2.1.1 Carbon monoxide rebreathing

**FIGURE 1:**
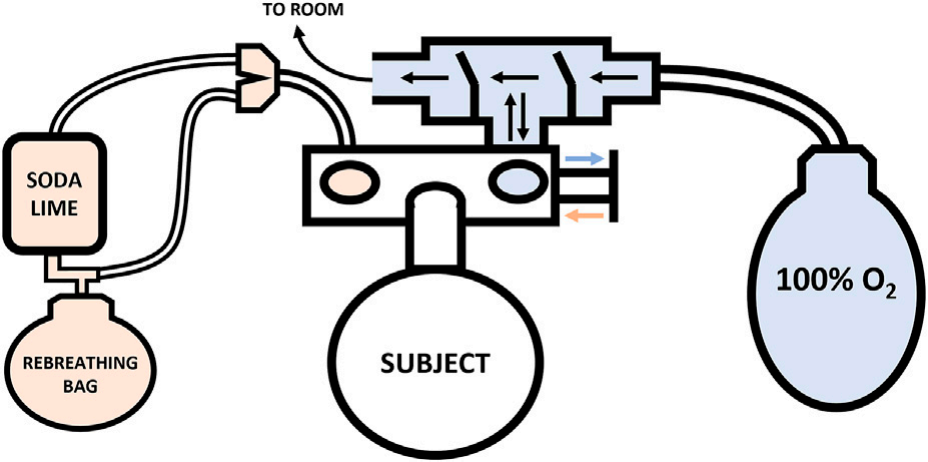
Schematic of the Hbmass CO rebreathing setup. Note: Closed-loop circuit—orange, Open-loop circuit—blue.

The assessment of Hbmass and PV was conducted according to the same CO rebreathing protocol and equations described in detail by Siebenmann et al. (2017). Briefly, participants entered the laboratory in a hydrated state, >2 h post-prandial, where their height (179.4 ± 7.0 cm) and weight (79.9 ± 10.0 kg) were immediately recorded. Participants consumed 500 ml of water before lying supine with their lower legs raised to facilitate complete blood mixing. Immediately thereafter, simultaneous baseline (BL) blood samples were drawn *via* venepuncture from an antecubital vein (4.5 ml lithium heparin vacutainer) and *via* the fingerstick procedure from the middle finger (100 µl). Prior to the collection of each capillary blood sample, the participants hand was pre-warmed for 5 min using a bespoke heating box. The participants remained in the same supine position, breathing ambient air for 20 min. During this period, a personalised dosage of 99.9% chemically pure CO (SIAD S. p.A., Bergamo, Italy) was prepared based on the participant’s weight (males: 1.5 ml kg^−1^; females: 1.2 ml kg^−1^) using a 140 ml syringe. Participants were then connected to an open-loop circuit (Figure 1; blue), where, they breathed 100% oxygen (O_2_) for 4 min to initiate denitrogenation of the lungs and other tissues, with expired air leaving the circuit into the room. Before transitioning to the closed-loop (Figure 1; orange), a second set of blood draws were taken and marked as pre-rebreathing (PRE). Following the blood draws, the participants fully exhaled before being switched to the closed-loop circuit by a sliding valve (Hans Rudolph Inc., Kansas, United States) to avoid overfilling the 6 L rebreathing bag (P3 Medical, Bristol, United Kingdom). Participants breathed 100% O_2_ within the closed-loop circuit for a 30 s period to allow them to settle and secure a good seal on the mouth piece. At the end of this period, their personalised dose of CO was introduced. The syringe was flushed three times while connected to the closed circuit to ensure complete delivery of the CO dose.

Participants rebreathed this gas mixture for 10 min. During the 10-min rebreathing period, exhaled carbon dioxide (CO_2_) was absorbed by soda lime within the circuit and one-way air valves ensured even mixing of the gas mixture and its proper movement through the system. Additional O_2_ was added to the closed-circuit *ad libitum* depending on the participants’ rate of O_2_ consumption. At the end of the 10-min rebreathing period, the participants again fully exhaled before being disconnected from the closed loop and connected to the open-loop circuit. Thus, all of the unabsorbed CO in the participant’s lungs was expired—except that in the residual volume—into the rebreathing bag for later analysis. Immediately after moving the participant to the open circuit, a final set of blood draws were completed and labelled post-rebreathing (POST).

The remaining gas in the closed-loop circuit was assessed for volume using a calibrated syringe (remaining gas +1.28 L circuit dead space) and a flow meter (S43OA Spirometric Module, KL Engineering Co., California, United States) and for CO concentration in parts per million (ppm) with a flue gas analyser (model DC710, TPI Europe Ltd., West Sussex, UK). The remaining unabsorbed CO in the closed-loop circuit was then calculated as a percentage of the original dosage.

##### 2.1.2 Blood analysis

All blood samples were immediately analysed using an ABL80 FLEX CO-OX Blood gas analyser (Radiometer Medical, Brønshøj, Denmark). The parameters of interest were haematocrit (Hct), haemoglobin concentration ([Hb]) and carboxyhaemoglobin concentration (COHb%). A single capillary tube containing 100 µl of blood could only be analysed once as 85 µl is required by the ABL 80. Venous blood samples were gently and thoroughly mixed for 30 s before being analysed in duplicate with the average used for the follow-up analysis. The following equations were used to calculate Hbmass, PV, BV and RBCV.

Haemoglobin mass was derived using the following set of equations in the following order:

$${nCO}_{absorbed}=P_{atm}\times {VCO}_{absorbed}÷\left(R\times T\right),$$ (1)

$${nHb}_{tagged}=\frac{{nCO}_{absorbed}}{4},$$ (2)

$${nHb}_{total}=\left(\frac{{nHb}_{tagged}}{∆COHb\%}\right)\times 100\%,$$ (3)

$${Hb}_{mass}={nHb}_{total}\times 6.44\times 10^{4}\;\frac{g}{mol},$$ (4)

where.

nCO_absorbed_ = number of CO molecules absorbed (mole).

P_atm_ = ambient pressure (standard atmospheres).

VCO_absorbed_ = volume of absorbed CO gas (L).

R = ideal gas constant (0.0821 L*atm/(mol*K).

T = temperature (K).

nHb_tagged_ = number of tagged haemoglobin molecules (mole).

nHb_total_ = number of total haemoglobin molecules (mole).

∆COHb% = change in carboxyhaemoglobin concentration (%).

6.44 × 10^4^ g/mol = molar mass of haemoglobin (g/mole).

Intravascular volumes (L) were determined through the following equations:

$$RBCV={Hb}_{mass}\times \frac{Hct}{Hbc},$$ (5)

$$BV=\frac{RBCV}{Hct},$$ (6)

PV=BV−RBCV, (7)

where.

RBCV = Red blood cell volume (L).

Hbmass = haemoglobin mass (g).

BV = Total blood volume (L).

PV = Plasma Volume (L).

Hct = Haematocrit (decimal value not %).

[Hb] = Haemoglobin concentration (g/L).

### 2.2 Experiment 2: Postural comparison

Fourteen adults (3 females, 11 males; height: 180.6 ± 6.7 cm, weight: 75.4 ± 8.3 kg, aged 21.0 ± 6.0 years) were included in a separate experiment designed to assess the effect posture (seated vs. supine with raised legs) may have on the following variables of interest [Hb], Hct and COHb%, which are used in the calculation of Hbmass. The same inclusion criteria were followed for both experiment 1 and 2. Additionally, the participants followed identical instructions to experiment 1 regarding food and liquid consumption prior to entering the laboratory. The participants were fully informed regarding the experimental procedures and then they provided written informed consent prior to having their height and weight recorded.

**FIGURE 2:**
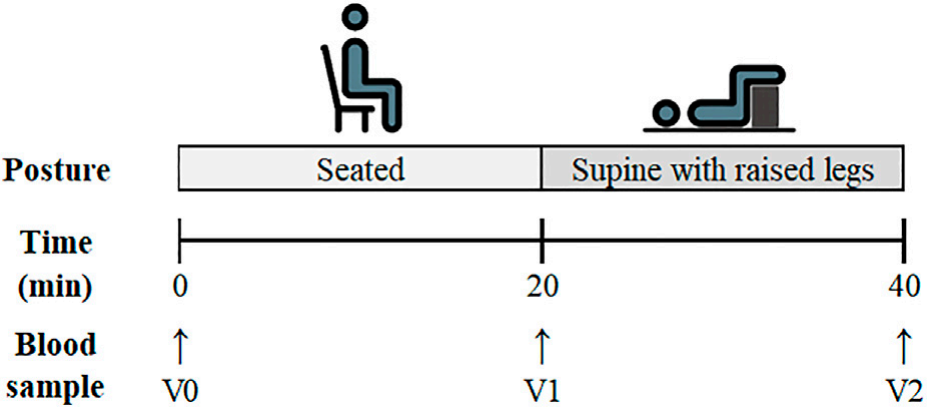
Schematic of the experimental protocol for Experiment 2: Postural Analysis. Venous blood sampling time points are listed as V0 (Pre-Seated), V1 (Post-Seated/Pre-Supine) and V2 (Post-Supine).

The participants were instructed to sit quietly and immediately upon sitting, a venous blood sample was drawn from an antecubital vein (marked as timepoint vein 0: V0) *via* venepuncture (4.5 ml lithium heparin vacutainer). They then remained seated for 20 min, at which point a second blood draw (time point vein 1: V1) was taken. The participants were subsequently moved to a supine position with their legs raised (identical to the resting supine position in experiment 1). The participants remained in this position for 20 min, after which a final blood sample was collected (time point vein 2: V2). The participants did not perform a CO rebreathing protocol after this postural comparison. A schematic detailing the experimental protocol is displayed in Figure 2.

### 2.3 Statistical analysis

Data are presented as Mean ± SD unless otherwise indicated. All statistical tests were performed unblinded to experimental conditions with SPSS (v25, IBM, New York, United States). GraphPad Prism (v8.4.2, GraphPad Software, San Diego, United States) was used for data visualisation. Significance level on all tests was set as *p* < 0.05, *a priori*. The paired *t-*test was used to identify differences between capillary and venous blood and the difference between BL and PRE time-points in certain analytes (Hct, [Hb], and COHb%). The between-variable (capillary vs. venous) relationship strength was calculated using Pearson’s correlation analysis and correlation coefficients were applied as recommended (Cohen, 2013; strong ≥0.60; moderate ≥0.40—<0.59; weak ≥0.20—<0.39). Intraclass correlation coefficient was utilised to assess test-retest correlation*.* The CO rebreathing typical measurement error (TE%) for each sampling site (venous or capillary) was calculated as a percentage of the mean value for each variable (Hopkins, 2000). Bland Altman plots (Bland and Altman, 1986) were used to compare venous and capillary blood samples and to compare Hbmass and PV from the two repeated tests as part of the repeatability analysis.

## 3 Results

**FIGURE 3:**
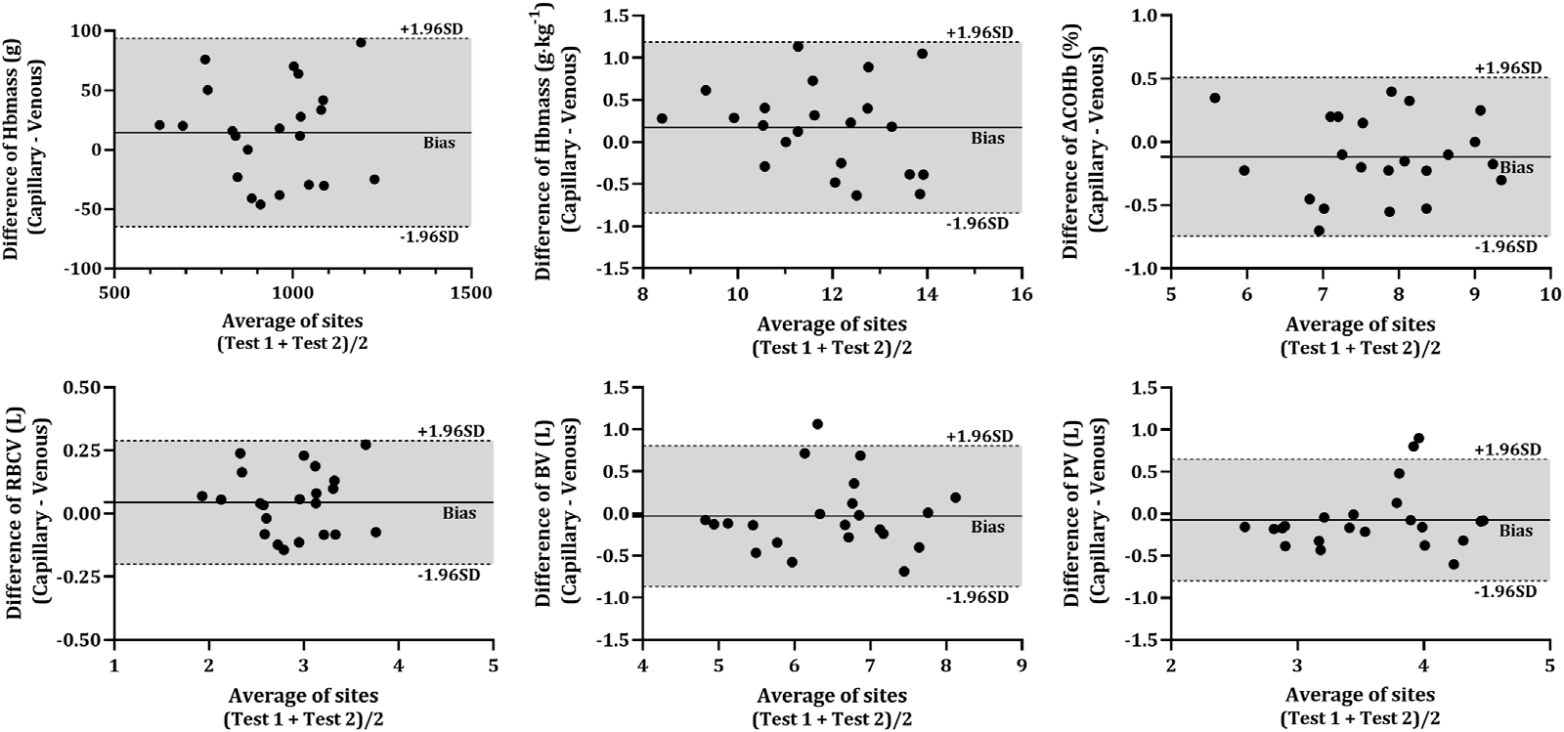
Difference in various analytes between capillary and venous blood after CO rebreathing. Horizontal lines indicate the mean bias (solid line) and the upper and lower 95% CI (dashed lines). Note: Average of sites—average of both sampling sites (VEN and CAP); Hbmass—haemoglobin mass; ΔCOHb%—change in carboxyhaemoglobin percentage; RBCV—red blood cell volume; BV—blood volume; PV—plasma volume.

As expected, given the low dose of CO inhaled, all participants completed the protocol with no signs of CO toxicity (highest COHb% = 10.65%). The analyte values determined from each blood sampling site (capillary and venous) and each time-point (BL, PRE and POST) are presented in Table 2. The Bland-Altman analysis (Figure 3) contain the individual data of analytes between capillary and venous blood. The mean unabsorbed CO percentage remaining in the circuit from the CO rebreathing protocol was 1.74 ± 0.42% (*cf.* Siebenmann et al. (2015); unabsorbed CO ranges from 0.8%–5.1%).

**TABLE 2:** Haematological variables determined from different sampling techniques (capillary and venous) during a CO rebreathing protocol. *N* = 22.

Analyte	Capillary		Venous		*t*-test	Correlation	
	Mean	SD	Mean	SD	*P*	*r*	*p*
BL Hct (%)	46.3	4.6	46.0	3.8	0.572	0.835	**<0.001**
Pre Hct (%)	45.3	4.5	44.3	3.5	0.058	0.860	**<0.001**
Post Hct (%)	45.6	4.0	44.3	4.0	**<0.001**	0.937	**<0.001**
BL [Hb] (g·L^−1^)	151	15	150	13	0.543	0.831	**<0.001**
Pre [Hb] (g·L^−1^)	148	15	145	11	0.061	0.863	**<0.001**
Post [Hb] (g.L^−1^)	149	13	144	13	**<0.001**	0.938	**<0.001**
BL COHb (%)	1.0	0.3	1.3	0.2	**<0.001**	0.238	0.143
Pre COHb (%)	0.8	0.2	1.2	0.2	**<0.001**	0.678	**<0.001**
Post COHb (%)	8.5	1.0	9.0	1.0	**<0.001**	0.947	**<0.001**
ΔCOHb (%)	7.7	1.0	7.8	1.0	0.101	0.950	**<0.001**
Hbmass (g)	948.8	156.8	943.4	157.3	0.108	0.967	**<0.001**
Hbmass (g·kg^−1^)	11.9	1.5	11.7	1.6	0.131	0.946	**<0.001**
RBCV (L)	2.9	0.5	2.9	0.5	0.109	0.966	**<0.001**
BV (L)	6.5	1.0	6.5	0.9	0.752	0.899	**<0.001**
PV (L)	3.6	0.6	3.6	0.6	0.360	0.816	**<0.001**

Note: BL, baseline; Hct, Haematocrit; [Hb], Haemoglobin concentration; COHb, Carboxyhaemoglobin; Hbmass, Haemoglobin mass; RBCV, red blood cell volume; BV, blood volume; PV, plasma volume, Bold = statistically significant (*p* < 0.05), *r* = Pearson’s correlation coefficient.

### 3.1 Validity

The COHb% obtained from capillary blood was significantly lower than that in venous blood at all time-points (*p* < 0.001). However, there were no significant differences present in the ΔCOHb% (*p* = 0.101) between CAP and VEN. All analytes obtained from capillary and venous blood were significantly correlated (*r* > 0.678, *p* < 0.001) except for COHb% at BL (*r* = 0.238, *p* = 0.143). No statistical differences were detected between any of the resultant Hbmass (absolute and relative) or intravascular volumes (RBCV, BV, and PV) calculated from venous or capillary blood (Table 2). The Bland-Altman analysis (Figure 3) further described the bias in the resultant absolute and relative Hbmass (absolute = 14.45 g LoA = −64.78 g—93.67 g; relative = 0.17 g kg^−1^, LoA = −0.84 g kg^−1^—1.19 g kg^−1^) and intravascular volumes (RBCV = 0.04 L, LoA = −0.20 L—0.29 L; BV = −0.03 L, LoA = −0.87 L—0.81 L; PV = −0.07 L, LoA = −0.80 L—0.65 L). The data dispersion within these plots is consistent irrespective of the site average, except in PV where values have greater dispersion over 3.78 L, although it is unclear why this dispersion is greater above this volume.

### 3.2 Experiment 2: Test-retest reliability

**FIGURE 4:**
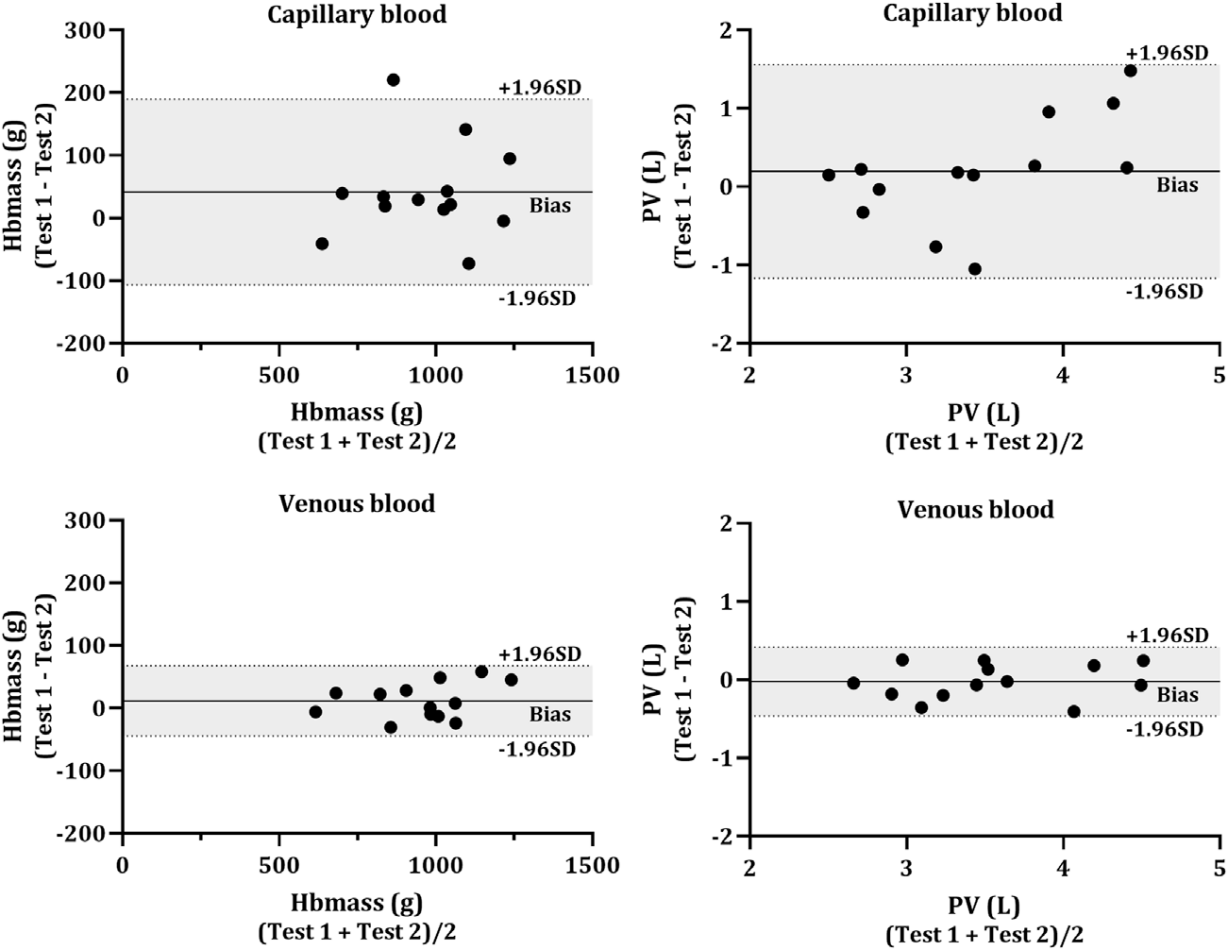
Bland-Altman plot for the test-retest reliability. Hbmass (left side) and plasma volume (PV; right side) calculated from capillary (top panels) and venous (bottom panels) blood. The individual data (*N* = 13) shows the difference between the Hbmass and PV determined from two CO rebreathing procedures. Horizontal lines indicate the mean bias (solid line) and the upper and lower 95% CI (dashed lines).

No significant differences were found in the change in mean (Test 2—Test 1) between capillary and venous blood for Hbmass (−41.4 g vs. −11.6 g, *p* = 0.160), relative Hbmass (−0.66 g kg^−1^ vs. −0.21 g kg^−1^, *p* = 0.145), ΔCOHb% (0.29% vs. 0.08%, *p* = 0.170), RBCV (−1.24 L vs. −0.04 L, *p* = 0.173), BV (−0.32 L vs. −0.15 L, *p* = 0.223) or PV (−0.19 L vs. 0.02 L, *p* = 0.277). Data for the TE% in each of the calculated variables are displayed in Table 3. Test-retest reliability, assessed through the intraclass correlation coefficient, other than COHb% before the inhalation of CO (BL and PRE) was strong in all analytes (Table 3). The Bland-Altman analyses (Figure 4) revealed a lower bias and bias SD in Hbmass and PV values from venous blood (Hbmass = 11.64 ± 28.60 g; PV = −0.02 ± 0.22 L) than capillary blood (Hbmass = 41.37 ± 75.70 g; PV = 0.19 ± 0.70 L).

**TABLE 3:** Reliability analysis of haematological variables determined from different sampling techniques (capillary and venous) during a CO rebreathing protocol. *N* = 13.

Analyte	Capillary			Venous			ICC	
	Mean	SD	TE%	Mean	SD	TE%	Capillary	Venous
BL Hct (%)	47.9	4.4		47.1	3.8		**0.888**	**0.949**
Pre Hct (%)	46.6	4.7		45.0	3.9		**0.843**	**0.950**
Post Hct (%)	46.9	4.2		45.4	4.2		**0.751**	**0.948**
BL [Hb] (g·L^−1^)	156	15		154	13		**0.891**	**0.963**
Pre [Hb] (g·L^−1^)	152	16		147	13		**0.842**	**0.946**
Post [Hb] (g.L^−1^)	153	14		148	14		**0.752**	**0.904**
BL COHb (%)	1.0	0.3		1.2	0.2		0.606	−0.196
Pre COHb (%)	0.8	0.3		1.2	0.3		−0.623	−1.132
Post COHb (%)	8.6	1.1		9.1	1.1		**0.976**	**0.969**
ΔCOHb (%)	7.8	1.2	4.9	7.9	1.2	1.9	**0.934**	**0.991**
Hbmass (g)	967.6	186.1	5.5	952.4	172.5	2.1	**0.949**	**0.993**
Hbmass (g·kg^−1^)	11.6	1.7	7.3	11.4	1.6	2.7	**0.827**	**0.978**
RBCV (L)	3.0	0.6	5.5	2.9	0.5	2.1	**0.949**	**0.993**
BV (L)	6.4	1.2	9.4	6.5	1.0	2.8	**0.844**	**0.986**
PV (L)	3.5	0.7	14.2	3.6	0.6	4.5	**0.732**	**0.968**

Note: Bold = statistically significant (*p* < 0.05); TE%, Typical measurement error %; ICC, intraclass correlation.

### 3.3 Postural comparison

**FIGURE 5:**
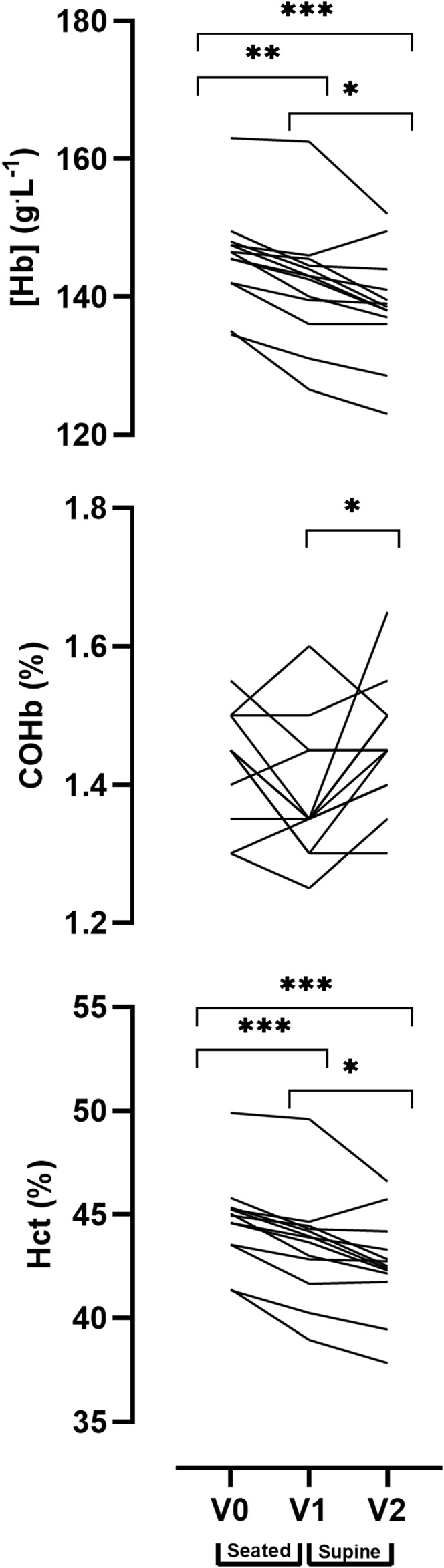
Effect of posture on venous blood values between three time points: Baseline (V0), 20 min seated (V1) and 20 min supine with legs elevated (V2). Data are displayed as individual values. Note: alpha level signified as * <0.05, ** <0.01, *** <0.001.

A one-way repeated measures ANOVA analysis revealed a significant main effect for time (V0, V1, and V2) in [Hb] (*p* < 0.001), COHb% (*p* = 0.021) and Hct (*p* < 0.001). Pairwise comparisons indicate a significant reduction in Hct between V0, V1 and V2 (V0 = 44.7 ± 2.1%, V1 = 43.4 ± 2.5%, V2 = 42.6 ± 2.3%) and reduced haemoglobin concentration [Hb] (V0 = 146 ± 7 g^.^L^−1^, V1 = 141 ± 9 g^.^L^−1^, V2 = 139 ± 8 g^.^L^−1^). The pairwise comparisons identified significant differences in the COHb% between V1 and V2 (i.e., sitting and supine, V1 = 1.38 ± 0.09%, V2 = 1.46 ± 0.09%), however, there were no significant differences between V0 and V1 or between V0 and V2. Individual responses to the postural analysis are presented for each timepoint in Figure 5.

## 4 Discussion

The present study’s primary aim was to assess the comparability of Hbmass and PV estimates derived from capillary or venous blood samples collected during a 10 min supine CO rebreathing protocol. The data demonstrate that capillary blood sampling is an acceptable alternative to venous blood for the calculation of Hbmass and blood vascular volumes (RBCV, BV, and PV) in terms of accuracy, even with a single sample analysis. Secondly, this study addressed whether posture plays a role in altering the variables of interest for the calculation of Hbmass ([Hb], Hct, and COHb%). There are significant reductions noted in the concentration of haemoglobin and haematocrit in a supine position compared to sitting. It is crucial to take into account the effect of posture and to interpret the data correctly based on the CO rebreathing protocol followed (Table 1). Finally, this study found reduced reliability and increased error compared to venous blood were present when using the capillary method, albeit limited. However, there is a methodological caveat that needs consideration, in particular, multiple replicate analyses of capillary blood samples, which were not carried out in the current study are a necessity in order to reduce the TE% from 5.5% to an acceptable level of 2%.

### 4.1 Validity

In contrast to previous research (Durussel et al., 2013), venous COHb% values were consistently larger and significantly different (Table 2) compared to those obtained from capillary blood across all time-points. Hütler et al. (2001), suggested that blood oxygen saturation could be a significant contributing factor to the COHB% measurement. However, the ΔCOHb% obtained from capillary and venous blood is statistically indifferent in the present study and shares a strong positive correlation. The difference noted between the two sampling sites is consistent and therefore allows for the accurate calculation of ΔCOHb% from either. Furthermore, despite a larger TE%, the current findings are in line with the literature, where the use of capillary or venous blood did not have any significant effect on the consequential calculations of absolute or relative Hbmass (Hütler et al., 2000; Gore et al., 2006; Prommer and Schmidt, 2007; Garvican et al., 2010).

Intravascular volumes (RBCV, BV, and PV) are calculated using Hct and [Hb] values. In the present study, no significant differences were noted between the Hct and [Hb] values collected at the PRE time-point between sampling techniques, however, POST [Hb] and Hct were significantly larger in the venous sample compared to in the capillary sample (Table 2). Although, one should bear in mind that these differences in POST values are of little consequence, as the calculation of Hbmass and PV only require the PRE time-point values. Indifferent, higher, or lower [Hb] and Hct values between venous and capillary sampling have been reported (Moe, 1970; Fahey and Rolph, 1975; Daae et al., 1988; Hütler et al., 2000). Such differences have been partially attributed to the capillary puncture depth, vessel diameter, sampling technique and blood flow rate to the extremities (Daae et al., 1988).

While there is ample evidence to support the use of a capillary sample as a valid method of blood collection during CO rebreathing, most evidence to date has been collected in a seated position (Burge and Skinner, 1995; Hütler et al., 2000; Prommer and Schmidt, 2007; Garvican et al., 2010). The overall accuracy of the capillary sampling method is as a result of replicate sample analysis in the range of 3–6 (Table 1). Although, there are some examples of publications with less replication (Steiner and Wehrlin, 2011; Durussel et al., 2013), no replication (Schmidt and Prommer, 2005), or simply do not state what type of analysis was conducted (Ashenden et al., 1999; Heinicke et al., 2001; Prommer and Schmidt, 2007; Otto et al., 2017). Finally, there is little evidence of the reliability of capillary sampling in a supine position during CO rebreathing (Kellenberger et al., 2022). The current study indicates that results obtained from a single capillary analysis were highly correlated with those from venous and that there were no significant differences between BL Hct and [Hb], ΔCOHb (%) or Hbmass calculated from venous or capillary samples. The capillary TE% is larger than typically noted in the literature, however, this error may most likely be a technical error associated with the ABL and can be confidently reduced through replicate analysis (Alexander et al., 2011). In doing so, it is certainly possible that the use of capillary sampling could be a valid tool/method for use in a supine CO rebreathing protocol.

### 4.2 Test-retest reliability

Intra-class correlation coefficients (ICC) for absolute and relative Hbmass from both blood sampling techniques indicated good to excellent reliability (>0.70) (Koo and Li, 2016). The intravascular volumes calculated from venous blood all had excellent ICC, while the values calculated with capillary blood ranged from moderate to excellent ICC (Table 3). When comparing CAP to VEN, the data indicates less reliable absolute and relative Hbmass determinations and intravascular volumes when calculated using blood drawn from the capillary. Values from venous blood consistently displayed higher ICC, lower TE%, and lower Bland-Altman bias (Table 3; Figure 4) when compared to capillary. The composition of the capillary blood sample and the technique used to collect it tend to result in a less reliable source of haematological variables. Capillary fingerstick samples often contain extracellular fluid which can potentially contaminate the blood sample readings. The smaller volume drawn through a single capillary sample both increases the impact of potential contamination and eliminates the option of duplicated analyses. Although as aforementioned, several authors have used other techniques than duplicate analysis, i.e., Durussel et al. (2013) collected duplicate samples and Steiner and Wehrlin (2011) collected triplicate from capillaries and averaged the singular analysis of those data to improve the accuracy of the method, while Hütler et al. (2000) averaged samples from separate time points. In other cases, increasing the size of the capillary tube may help. Gore et al. (2006) collected capillary samples up to 200 µl to allow for five replicate analyses of each time point. This would not be a suitable volume for quintuplet analyses with the ABL 80 which requires 85 µl per capillary sample.

Reliable capillary blood sampling is dependent on maintained blood flow to the sampling site (ear lobe or finger) which will reduce drop-to-drop variability and error (Bond and Richards-Kortum, 2015). Over manipulation or “milking” of the finger to extract blood from participants with a lower blood flow during the sampling time can increase the drop-to-drop variability and dilute the sample with interstitial fluid. Further consideration should be given to the effects of deep lancing and haemolysation of the sample (Cembrowski and Füzéry, 2017). Additionally, due to the participants’ posture during the CO rebreathing protocol (supine, legs raised), the current researchers found blood flow was reduced in most cases, despite limb warming and the pre-test consumption of 500 ml of water. Some participants’ capillary blood samples could not be included in the analysis, as the sample clotted in the capillary tube as a result of the blood sample collection taking excessive time. Thus, reinforcing the essential use of heparinised capillary tubes.

Siebenmann et al. (2017) states that a TE% of less than 2.5% has been common in recent CO rebreathing studies and that experts with this method often report TE% of 1.5%–2%. The TE% of 2.1% reported from venous blood in the current study is comparable with the literature assessing CO rebreathing protocol reliability (Table 1 provides details of published TE% and CoV: (Burge and Skinner, 1995; Hütler et al., 2000; Schmidt and Prommer, 2005; Kellenberger et al., 2022; Oberholzer et al., 2020; Siebenmann et al., 2017). However, the TE% of 5.5% for Hbmass calculated from capillary blood is larger than previously reported, although there are examples of similar or higher TE%. Oberholzer et al. (2020) reported (in venous) TE% of up to 14.1% caused by changes in CO dose, length of rebreathing, number of replicates and the altitude that the protocol was carried out at. In order to ensure complete mixing—of CO & blood—and thus tagging of the haemoglobin molecule with CO both Garvican et al. (2010) and Siebenmann et al. (2017) suggest that 10 min is an appropriate amount of time after the onset of CO rebreathing to draw the post sample. This is further substantiated by Kellenberger et al. (2022) who reports similar COHb% values for capillary and venous samples at 8 and 10 min of rebreathing. Unfortunately, Kellenberger et al. (2022) did not conduct a similar repeatability analysis utilised in the current study of CAP and VEN values using the Siebenmann method.

### 4.3 Postural comparison

A seated posture is often used in alternate versions of CO rebreathing protocols (Hütler et al., 2000; Prommer and Schmidt, 2007; Durussel et al., 2013), however, the protocols described by Siebenmann et al. (2017) and Burge and Skinner (1995) are conducted in the supine position, either with or without legs raised respectively. The change of posture from seated to supine allows the protocol to be used during bed rest interventions where participants are required to remain horizontal which is useful in both clinical and space life science settings. Raising the legs also increases whole body blood circulation, reduces the risk of syncope and allows for thorough distribution of the CO bolus throughout the circulation. Further, performing the protocol in a seated position is reported to lead to under-estimations of Hbmass of approximately 28 g (∼3%) (Keiser et al., 2013; Siebenmann et al., 2017). Data from Experiment 2 (Figure 5) highlights that there is a postural influence (seated vs. supine with legs raised) on [Hb], Hct and COHb%; and caution should be exercised when calculating and comparing Hbmass between protocols. Further, Lima-Oliveira et al. (2017) report clinically significant shifts in [Hb], Hct and PV as a result of changing posture after 20 or 25 min in a seated or supine position. In the present study, participants lay supine with their legs raised for 20 min prior to the 4-min O_2_ pre-breathe. The purpose of this period was to allow for increased blood circulation from the lower to upper extremities in order to reduce blood pooling in the legs which may result in PV shifts (Diaz et al., 1979). Changing the participants’ position from supine to standing results in significant BV redistribution and erroneous calculation of PV of up to 500 ml (Hagan et al., 1978).

In contrast with the current results (Figure 5), Durussel et al. (2013) discovered no postural effect (seated vs. supine) on the estimation of Hbmass, BV, RBCV or PV. However, it is important to note that the authors conducted only 10 min in the supine position followed by 10 min in the seated position when 20 min in one posture is suggested for adequate stabilisation (Tan et al., 1973; Smith et al., 1994). Therefore, the protocol used in their study neither waited long enough for vascular volumes to stabilise, nor counter the effect of the supine position on the seated position in order to draw the conclusion that posture does not affect Hbmass calculation. Finally, by obtaining blood samples from an antecubital vein and the saphena magma of the ankle, Keiser et al. (2013) found there to be an uneven distribution of CO in the blood ciruculation if the CO rebreathing protocol is performed in the seated position. This uneven distribution was ascribed to incomplete mixing of CO and may be negated by either gently exercising or by conducting the protocol in a supine position (Siebenmann et al., 2017).

### 4.4 Practical implications

Based on the current data, several recommendations may be put forward for consideration when using the current implemented rebreathing protocol. As aforementioned, a 20-min rest in a supine position with legs raised is essential for conducting this protocol. Failure to allow for this period may result in significantly higher values of [Hb] and Hct in the Pre-rebreathing data than if there had been a stabilisation period following the posture change (Figure 5).

Venous sampling is typically preferred when performing the methods described in the present study (Siebenmann et al., 2017; Rønnestad et al., 2021). However, in case a phlebotomist is unavailable, a capillary blood sample may be obtained instead. Previous evidence indicates that it is highly recommended to collect capillary samples large enough to be analysed in quadruplicate (Gore et al., 2006; Alexander et al., 2011). Failing that, several samples should be drawn with the singular analysis of each being averaged.

Capillary tube filling time of approximately 20 s have been reported (Gore et al., 2006; Durussel et al., 2013) and as previously mentioned, this was not the case in the current study and several samples clotted, requiring that they be discarded. As such it is highly recommended to use heparinised capillary tubes when conducting the study in a supine position to avoid any clotting related issue prior to analysis and thus loss of data.

In the protocol described by Schmidt and Prommer (2005), CO in the lung pre- and post-rebreathing is calculated and accounted for. This correction means an increased precision in the measurement and reduction in potential typical error of measurement of ∼4 g when assuming an absolute Hbmass of 950 g (<0.5%) (Prommer and Schmidt, 2007) and should be used, in combination with other mentioned factors. Further, it should be considered that with prolonged CO rebreathing there is an increase in the loss of CO from the vasculature, this CO may become bound to myoglobin. In order to reduce error by as much as possible, these corrections may be applied.

Finally, when using the capillary or venous sampling site for the estimation of Hbmass and blood volumes, the sampling sites must not be used interchangeably during testing. For example, if a venous line coagulates during the waiting period between samples, a new venous line should be inserted or venepuncture performed rather than switching to drawing capillary blood, or *vice versa*. The reasoning for this suggestion is that there was a significant difference of 0.5% for the COHb% values between sampling sites. Failing to account for this consistent error between the methods would lead to an error of ∼50 g based on an absolute Hbmass of 950 g as reported for the current participants.

### 4.5 Limitations

As noted in the Postural comparison section, the supine posture with elevated legs is the preferred position for measuring Hbmass. Blood collection from an antecubital vein was not affected whilst supine, however, in our laboratory, we found that fingertip capillary draws had reduced blood flow. Consequently, fingertip blood draws often took longer, clotted or were unsuccessful. Increased duration may also have affected drop to drop variation and ultimately the parameters of interest. As a result, large capillary samples were not obtained and the results were analysed in singular. Assessing the capillary sample in duplicate may reduce the error by a factor of 2 (Schmidt and Prommer, 2005), a comparable reduction in error may be seen in our results (Table 2) between venous (2.1%) and capillary (4.9%) samples.

The main limitation in the comparison of capillary and venous blood data in the present study is the inability to collect sufficiently large capillary samples while the participant lay in the supine posture, despite taking several measures to increase the ease of the blood draw. To address issues that others may face in these circumstances, the following is recommended: 1) 10 min of hand warming be conducted prior to capillary blood draws, 2) use of heparinised capillary tubes, 3) collect sufficiently large capillary blood samples that allows for the assessment of all samples in at least duplicate. Further, based on the literature presented in Table 1, replicate analysis in the range of 3–6 times is highly recommended.

## 5 Conclusion

The calculated measures of absolute and relative Hbmass, and intravascular volumes, are considered valid when determined using capillary drawn blood due to the statistical indifference and high correlation with values calculated from venous drawn blood. Reliability analysis found that ICC and change of mean values are considered acceptable for capillary blood use. Higher TE% indicates a certain level of caution should be applied when using capillary sampling analysed in the singular for research or medical purposes. Finally, significant differences between the seated and supine (V0, V1, and V2) blood values indicate the importance of a 20-min supine rest with legs raised before the collection of the PRE sample.

## Data Availability

The raw data supporting the conclusion of this article will be made available by the authors, without undue reservation.
